# Thymidine kinase 1 related to Prolif-like T cells promoted GBM through regulation of cell cycle and EMT signals: a comprehensive research based on multi-omics analysis and experimental validation

**DOI:** 10.3389/fimmu.2025.1655980

**Published:** 2025-09-25

**Authors:** Wentao Deng, Xiaoting Chang, Wei Shang

**Affiliations:** ^1^ Department of Neurosurgery, First Affiliated Hospital of Dalian Medical University, Dalian, Liaoning, China; ^2^ Department of Neurosurgery, Panjin Central Hospital, Panjin, Liaoning, China; ^3^ Department of Neurology, Second Affiliated Hospital of Dalian Medical University, Dalian, Liaoning, China

**Keywords:** glioblastoma, thymidine kinase 1, Prolif-like T cell, scRNA-seq, stRNA-seq

## Abstract

**Background:**

Glioblastoma (GBM) is a common high-grade glioma characterized by a significantly immuno-suppressed immune microenvironment. Prolif-like T cells are a subset of T cells whose expression of related markers can influence the tumor microenvironment.

**Methods:**

This study used scRNA-seq and stRNA-seq to identify markers associated with Prolif-like T cells in the GBM tumor microenvironment. Survival analysis and consistency clustering were then employed to identify GBM subtypes associated with Prolif-like T cells, followed by an analysis of differences between subtypes. This study constructed survival models and scRNA-seq to screen for important genes associated with Prolif-like T cells in GBM and further investigated the role of TK1 in the cell cycle and EMT processes of GBM.

**Results:**

Using scRNA-seq from 149002 GBM cells, our study identified 593 Prolif-like T cell-related markers. The results of stRNA-seq revealed the close association of Prolif-like T cell with cell cycle and EMT signals. In addition, 82 genes were found to influence GBM prognosis. Based on the expression of the 82 genes, two Prolif-like T cell-related GBM subtypes (C1 and C2) were constructed, with C1 exhibiting stronger proliferative activity. Survival models and scRNA-seq identified TK1 as a key gene associated with Prolif-like T cells in GBM. Further studies revealed that TK1 promotes GBM progression by influencing cell cycle and EMT processes, and targeting TK1 inhibition suppresses GBM proliferation and migration.

**Conclusions:**

TK1, as a Prolif-like T cell-associated marker, promotes GBM progression and can serve as a potential therapeutic target for GBM.

## Introduction

Glioblastoma (GBM) is the most common and most aggressive primary malignant brain tumor in adults, classified as Grade IV in the glioma classification system. It accounts for over 54% of all glioma cases annually (1). This tumor originates from astrocytes and is characterized by high heterogeneity, rapid proliferation, and diffuse infiltrative growth, often involving the deep white matter of the cerebral hemispheres, particularly the frontal and temporal lobes. Molecular pathologically, over 90% of glioblastomas are IDH wild-type, with characteristic molecular alterations including chromosome 7 amplification/10 deletion, TERT promoter mutations, EGFR amplification, and PTEN mutations (2). Clinical symptoms are primarily caused by tumor mass effect and increased intracranial pressure, including progressively worsening headaches, seizures, focal neurological deficits, and cognitive decline, with a typical disease course of less than 3 months. Treatment follows a surgery-based comprehensive strategy: maximal safe resection is the primary goal, followed by standard postoperative treatment with concurrent chemoradiotherapy, followed by six cycles of adjuvant temozolomide. Despite this, tumor recurrence is nearly inevitable, with a median progression-free survival of 6.9–7.5 months and a median overall survival of approximately 15–18 months (3). Immune checkpoint inhibitors (such as nivolumab) have limited efficacy in GBM, potentially due to the tumor-associated immuno-suppressive microenvironment (4). Exploring the cellular and molecular changes in the immune microenvironment of GBM remains a critical research direction for GBM treatment.

In recent years, immune therapy strategies targeting T cells have shown promise, but the unique immuno-suppressive microenvironment of GBM severely limits T cell antitumor activity. The GBM tumor microenvironment (TME) is characterized by significant immune suppression, manifested as insufficient T cell infiltration and severely impaired function. TGF-β is highly expressed in the GBM TME, not only inhibiting T cell proliferation and effector functions but also promoting the expansion of regulatory T cells (Tregs), further suppressing antitumor immunity (5). Additionally, elevated activity of the key tryptophan metabolism enzyme IDO1 in GBM leads to local tryptophan depletion and accumulation of toxic metabolites, which suppress T cell activity and promote Treg differentiation (6). Myeloid-derived suppressor cells (MDSCs) are highly infiltrated in GBM and suppress T cell function by producing arginase, reactive oxygen species, and nitric oxide (7). Notably, even when T cells are infiltrated in the GBM TME, their functional state is often in an “exhausted” state, characterized by sustained high expression of multiple inhibitory receptors (PD-1, TIM-3, and LAG-3) and reduced ability to produce effector cytokines (IFN-γ and TNF-α) (8). Prolif-like T cells are a unique subset of exhausted precursor T cells capable of both generating new effector T cells and terminating into terminally exhausted T cells. The emergence of this cell population may mark the onset of T cell exhaustion and has recently been identified as a key regulatory node in antitumor immune responses and a decisive factor in immune checkpoint blockade therapy response (9). However, the role of this T cell subset in the TME of GBM remains unclear. Therefore, this study aims to identify Prolif-like T cell-associated markers in GBM using single-cell transcriptomics and further investigate the effects of key markers on cellular functions and immune microenvironment changes in GBM, with the goal of determining whether Prolif-like T cell-associated markers can serve as potential therapeutic targets for GBM.

## Materials and methods

### Single-cell RNA sequencing data sources and standardization

In this study, we collected six scRNA-seq data cohorts of GBM from the GEO database. These cohorts are GSE103224 (10), GSE138794 (11), GSE141383 (12), GSE162631 (13), GSE223063 (14, 15), and GSE235676 (16), comprising a total of 43 GBM samples. These datasets were read using the Seurat package (min.cells = 3, min.features = 200) and underwent quality control (nCount_RNA ≥ 1000, nFeature_RNA: 200–8000, percent.mt ≤ 20), followed by merging into a scRNA-seq dataset (17). The dataset contains a total of 149,002 cells, with each cell undergoing mRNA detection for 45,131 genes.

### Cell clustering and annotation

The merged scRNA-seq dataset was normalized using the LogNormalize method, and 2,000 highly variable genes were identified and normalized (18). PCA dimensionality reduction analysis was performed on the dataset based on the highly variable genes, and batch effects were removed (19). The datasets were clustered using the FindClusters method, resulting in 52 cell types (numbered 0-51). The 52 cell types were manually annotated based on known cell marker genes, resulting in 12 major cell clusters and an unknown cell cluster. Using the findmarker function, identify cell subtype-specific markers (only.pos=T, min.pct=0.25, logfc.threshold=0.25), and further obtain proliferation-related markers associated with Prolif-like T cells (log2fc > 0.585), yielding a total of 593 genes.

### Cell communication analysis and pseudo-time analysis

This study applied the CellChat method (20) and CellPhoneDB data to analyze cell communication pathways associated with Prolif-like T cells in scRNA-seq data, identifying cell communication pathways related to Prolif-like T cells. Additionally, the Monocle method was used for pseudo-time series analysis to examine the expression changes of proliferative markers associated with Prolif-like T cells during the development of tumor cells and Prolif-like T cells.

### stRNA-seq analysis

The CreateSeuratObject and Read10X_Image functions were used to load spatial transcriptomics data (GSE253080) (21), while the SCTransform function was applied for data normalization. To evaluate various cellular features such as proliferative T cell signatures, cell cycle activity, EMT characteristics, and tumor cell traits, we employed five scoring algorithms: Add, AUCell, UCell, ssGSEA, and singscore (22). To minimize potential biases from individual methods, we aggregated the results from all five algorithms to generate a composite score, referred to as the Scoring, which we considered the true feature score.

### RNA-seq data sources and standardization

This study obtained five GBM transcriptomic cohorts with clinical data from the TCGA, CGGA, and GEO databases. The data from TCGA included 160 patients, while the data from CGGA included 216 patients(CGGA325, CGGA133), and the GEO dataset includes 120 patients (GSE7695, GSE83300). The five datasets were merged and batches were removed via “sva” package (23), resulting in a transcriptomic matrix containing 496 GBM samples for subsequent gene screening and functional analysis.

### Survival analysis and gene screening

In this study, we performed gene screening of 593 Prolif-like T cell-related markers obtained from single-cell data using transcriptomic data and univariate Cox analysis. We further screened out 82 key genes with high-risk characteristics and significant association with prognosis in GBM. Based on the expression levels of the 82 key genes and ssGSEA analysis, we further obtained the pathway enrichment scores related to Prolif-like T cells in GBM patients to evaluate the enrichment level of Prolif-like T cells in GBM patients. KM analysis was used to detect the effect of pathway enrichment scores on the prognosis of GBM patients.

### GBM subtypes and differential features

In this study, based on the ConsensusClusterPlus package (24, 25) and 82 key genes, we performed consensus clustering to explore GBM subtypes associated with Prolif-like T cells, identifying two subtypes, C1 and C2. We then conducted differential analysis and pathway enrichment analysis on the two subtypes. Pathway enrichment analysis was performed using the ssGSEA analysis method and the Hallmark pathway set (from the MSigDB database) to analyze the differences in pathway activity between the two subtypes. Based on the oncoPredict package (26), drug sensitivity prediction was performed for the two subtypes to distinguish their sensitivity to common chemotherapy drugs.

### Immune infiltration analysis

This study first used the ESTIMATE algorithm to calculate the tumor purity, stroma score, and immune score of the two subtypes to analyze the differences in the immune microenvironment of the two subtypes as a whole. Then, the TIMER database was used to analyze the immune cell infiltration levels and immune checkpoint changes of the two subtypes to further analyze the differences in the immune microenvironment of the two subtypes.

### Survival model construction and validation

In order to further screen out important genes with survival prediction ability from 82 key genes, a survival prediction model was constructed using the survival data of 496 patients. Among them, the data from TCGA and GEO were used as the internal dataset, while the data from CGGA were used as the external dataset. The training dataset (train) was generated by randomly splitting the internal dataset into 50%, while the test dataset 1 (test1) was the remaining part of the internal dataset, and the test dataset 2 (test2) was the entire internal dataset. The test dataset 3 (test3) was the entire external dataset. Lasso analysis was performed on the 82 key gene data in the training dataset, and a Cox regression model was further constructed. Three important genes with survival prediction capabilities were ultimately screened and validated using the three test sets.

### Gene function analysis of TK1

This study first analyzed the function of the TK1 gene in GBM based on the BEST (Biomarker Exploration of Solid Tumors) online tool (27). The main analysis focused on the expression changes of the TK1 gene in GBM and its impact on the prognosis of GBM patients. Further analysis was conducted on the association between the TK1 gene and common proliferation markers and related enriched pathways. Finally, the SNV mutations and mRNA expression of TK1 in the TCGA pan-cancer dataset were analyzed.

### Cell culture

This study also used two cell lines, U251 and U87. U251 cell was cultured by a combination of DMEM and FBS; however, U87 cell was cultured by a combination of MEM and FBS.

### Cell transfection

In order to explore the role of TK1 on the proliferation of GBM cells, we applied knock-down experiments via siRNA technologies. The detailed processing of knock-down experiments was carried out strictly according to the siRNA-Mate Plus Transfection Kit protocol provided by GenePharma. The siRNA-TK1, siRNA-NC, and other related products were provided by Huzhou Hippo Biotechnology Co., Ltd. The specific interference sequences are as follows: siRNA1 (sense: UGUCGGCUCUGCUACUUCAAGTT; antisense: CUUGAAGUAGCAGAGCCGACATT), siRNA2 (sense: CCAAAGACACUCGCUACAGCATT; antisense: UGCUGUAGCGAGUGUCUUUGGTT), siRNA3 (sense: CGUGGCUGUCAUAGGCAUCGATT; antisense: UCGAUGCCUAUGACAGCCACGTT). Finally, the knockdown efficiency was assessed 48 h after transfection. The primer sequences for TK1 are as follows: F-GGGCGTGGCTGTCATAGGC, R-GCGGCACCAGGTTCAGGATG.

### CCK8 experiment

The CCK-8 kit was provided by Meilunbio. The successfully transfected cells were trypsinized and seeded into 96-well plates, with approximately 100 μL of cell suspension per well. At 24 h, 48 h, and 72 h after seeding, CCK-8 solution was added to each well at a final concentration of 10% for detection, and absorbance was quantified at 450 nm using a microplate reader.

### Migration and invasion assays

In order to explore the role of TK1 on the migration and invasion of GBM cells, we applied scratch experiments and transwell invasion experiments. Cells transfected with siRNA were seeded into 6-well plates at an appropriate density. Once they reached confluence, a scratch was made using a 200 μL pipette tip to assess cell migration. Similarly, cells at an appropriate density were seeded into transwell chambers, where invasion was induced by culture medium containing a high concentration of serum. The effect of TK1 knockdown on this process was then evaluated. Cells were observed and photographed under a microscope after 24 h.

### Western blot

Cellular proteins were extracted using lysis buffer supplemented with PMSF, protease inhibitors, and phosphatase inhibitors. After adding an appropriate amount of loading buffer, the samples were boiled. Proteins were separated using Precast Protein Plus Gel (YEASEN, 36266ES10) and Precast Running Buffer (Tris-Mops) (YEASEN, 36271ES05) at 120V for 50 minutes. Subsequently, membrane transfer was performed using fast transfer buffer (Servicebio, G2028-1L). The membrane was then blocked with 5% skim milk at room temperature for 1 hour, followed by overnight incubation with primary antibodies: TK1 (Proteintech, 15691-1-AP, 1:5000), E-cadherin (Proteintech, 20874-1-AP, 1:20000), N-cadherin (Proteintech, 22018-1-AP, 1:4000), Vimentin (Proteintech, 10366-1-AP, 1:20000), Cyclin A1 (Proteintech, 13295-1-AP, 1:500), Cyclin B1 (Proteintech, 55004-1-AP, 1:1000), and β-Actin (ABclonal, AC043, 1:5000). On the next day, the membrane was incubated with secondary antibodies at room temperature for 1 hour before chemiluminescent detection.

## Results

### Screening of Prolif-like T cell-related markers using RNA-seq and scRNA-seq

**Figure 1:**
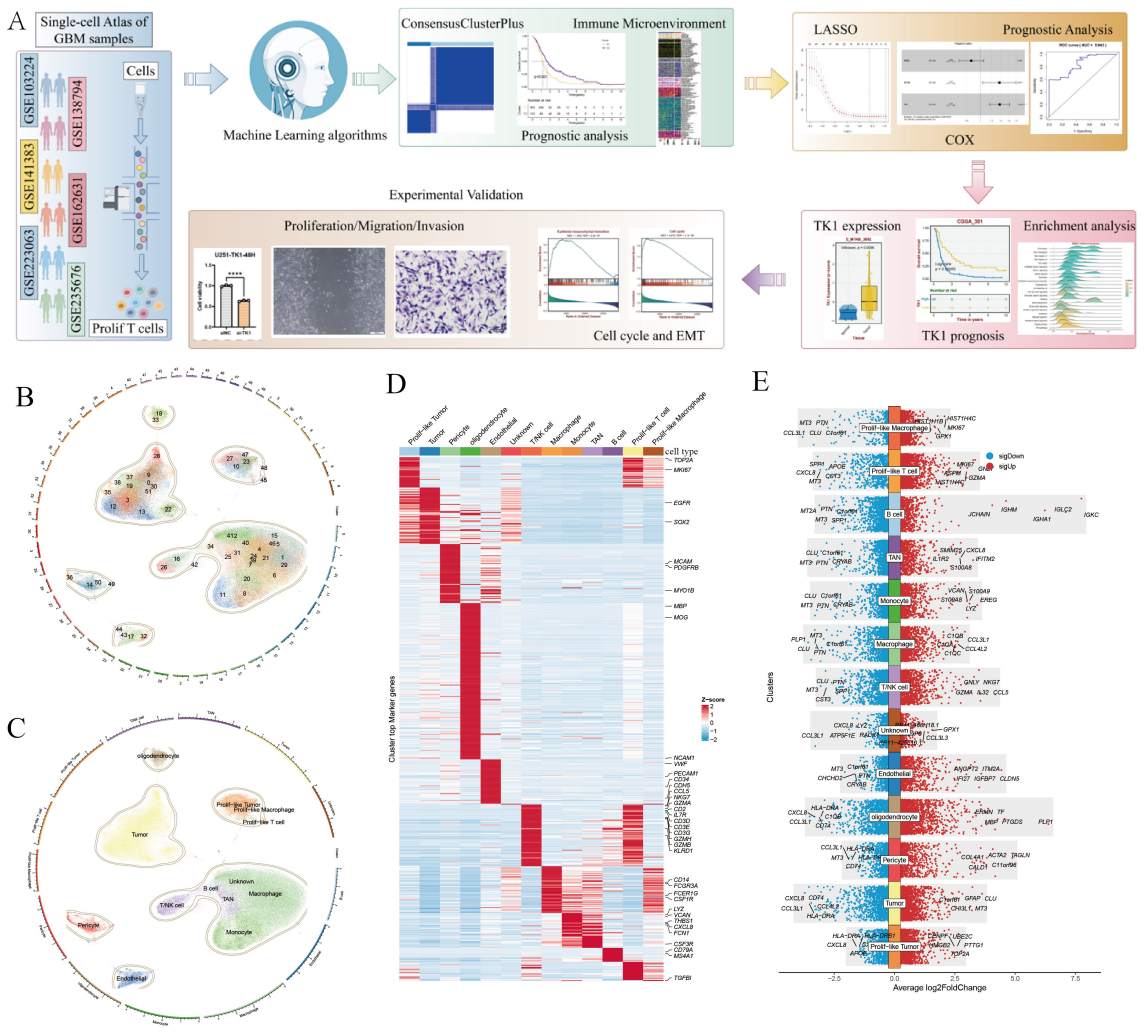
Screening of markers associated with Prolif-like T cells. **(A)** Analysis workflow. **(B)** 52 cell clusters identified by scRNA-seq. **(C)** 12 cell types identified by scRNA-seq. **(D)** Heat map of cell-specific marker expression in 12 cell types. **(E)** Bar chart of cell-specific marker expression in 12 cell types. (The workflow diagram in **(A)** of this study was created using Figdraw and has been approved, with the authorization code as follows: TYUSWc04c0).

This study integrated RNA-seq data and scRNA-seq data from a large-scale analysis of 496 patients and 149002 cells with GBM multiforme (**Figure 1A**). This study assumes that the tumor tissue extracted from RNA-seq data is a mixture of multiple cell types, and the gene expression represents the overall expression level of that gene across multiple cell types in the scRNA-seq data, reflecting the overall genetic changes in the tumor tissue. We performed single-cell clustering and annotation on 149002 cells, identifying 52 cell types (**Figure 1B**) and 12 cell subpopulations (**Figure 1C**). The 12 cell subpopulations are TAN, endothelial, pericyte, monocyte, oligodendrocyte, tumor, macrophage, prolif-like tumor, prolif-like macrophage, prolif-like T cell, T/NK cell, and B cell. This study found that prolif-like T cells not only express T cell surface markers (CD3D and CD3E) but also highly express proliferation-related markers such as TOP2A and MKI67 (**Figure 1D**). Additionally, these proliferation markers are also up-regulated in prolif-like tumor and prolif-like macrophage (**Figure 1E**). This suggests that these proliferative markers associated with prolif-like T cells may be important characteristics of GBM tumor tissues.

**Figure 2:**
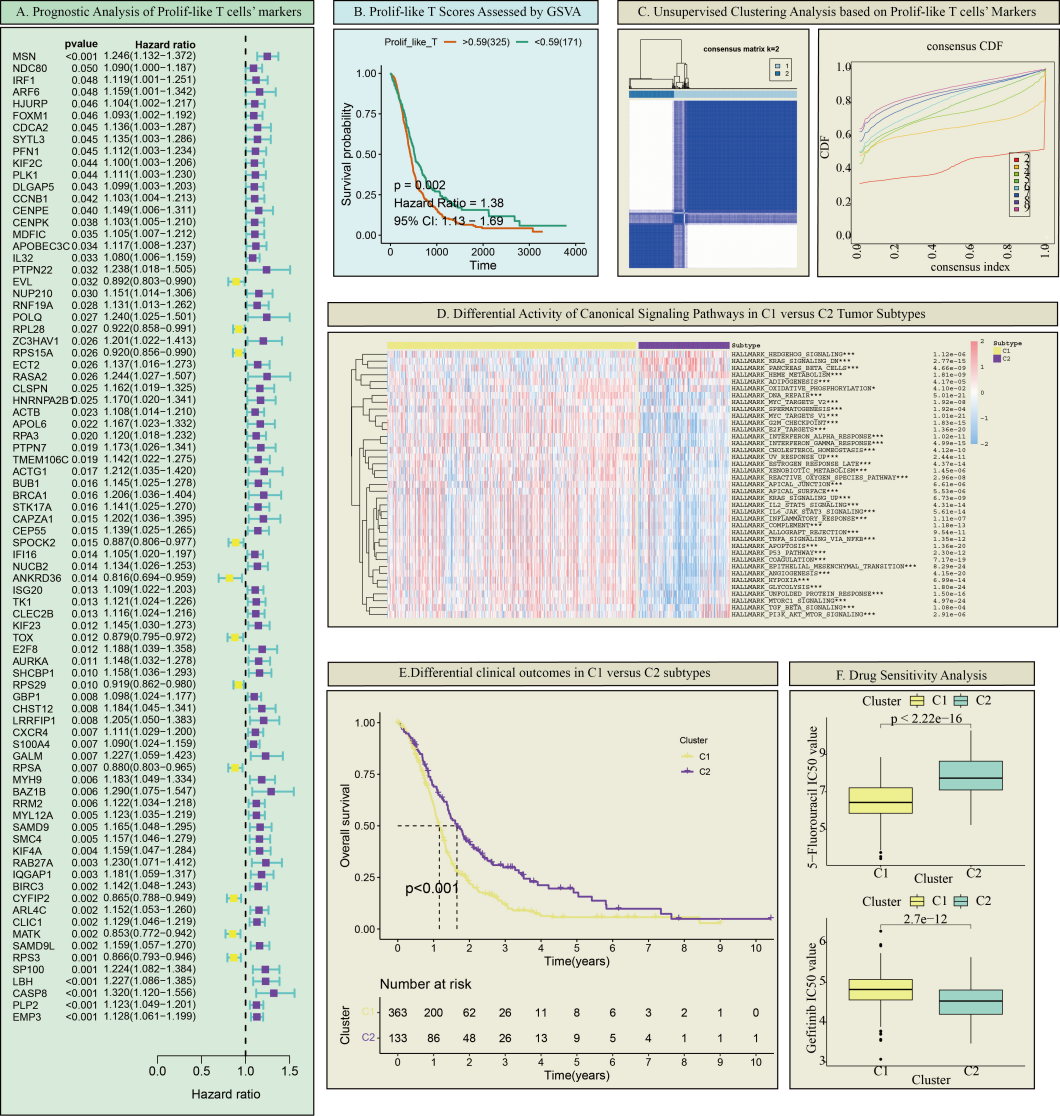
Prolif-like T cell-associated GBM subtypes. **(A)** Prognostic analysis of Prolif-like T cell markers. **(B)** Prolif-like T scores assessed by GSVA. **(C)** Unsupervised clustering analysis based on Prolif-like T cell markers. **(D)** Differential activity of canonical signaling pathways in C1 versus C2 tumor subtypes. **(E)** Differential clinical outcomes in C1 versus C2 subtypes. **(F)** Drug Sensitivity Analysis. (*p < 0.05; ***p<0.001).

This study further identified markers associated with prolif-like T cells, totaling 2,261 genes. Using the criteria of Log2FC > 0.585 (fold > 1.5), 2,261 genes were screened, resulting in 593 significantly differentially expressed genes. Based on transcriptomic survival data, single-factor Cox regression analysis identified 82 key genes significantly associated with GBM survival prognosis (**Figure 2A**). Most of these genes are high-risk genes (HR > 1) associated with GBM survival. This study suggests that the overall status of these 82 genes can reflect the enrichment level of prolif-like T cells in GBM patients and have certain research values. Therefore, this study used the ssGSEA method in the GSVA package to perform pathway enrichment scoring of the expression of 82 genes in the GBM transcriptome. This score reflects the degree of prolif-like T cell enrichment in GBM patients. Based on KM survival analysis, the optimal cutoff value was automatically determined. This study found that patients in the high-score group (325 cases) had significantly poorer survival prognosis than those in the low-score group (171 cases), suggesting that increased prolif-like T cell enrichment is an indicator of poor prognosis in GBM patients (**Figure 2B**).

### Differences in GBM subtypes and immune microenvironments associated with Prolif-like T cells

This study found that the expression of 82 markers associated with prolif-like T cells, obtained from scRNA-seq data, had a certain influence on the clinical prognosis of GBM. This suggests that these marker genes may effectively distinguish the status of GBM patients. Therefore, this study performed consistency clustering based on the expression of 82 key genes, dividing 496 GBM patients into two categories, C1 and C2 (**Figure 2C**). Using ssGSEA analysis and the Hallmark pathway dataset, we analyzed the enrichment levels of common pathways in the two categories. The results showed that C1 exhibited increased enrichment of proliferative pathways (such as the KRAS pathway, AKT pathway, and Hedgehog pathway) compared to C2 (**Figure 2D**). KM survival analysis revealed that C1 had shorter survival times and poorer prognosis compared to C2 (**Figure 2E**). Drug sensitivity analysis showed that C1 was more sensitive to 5-fluorouracil than C2, while C2 was more sensitive to gefitinib than C1 (**Figure 2F**). In summary, C1 exhibited more active proliferative activity than C2 and represents a more malignant GBM subtype.

**Figure 3:**
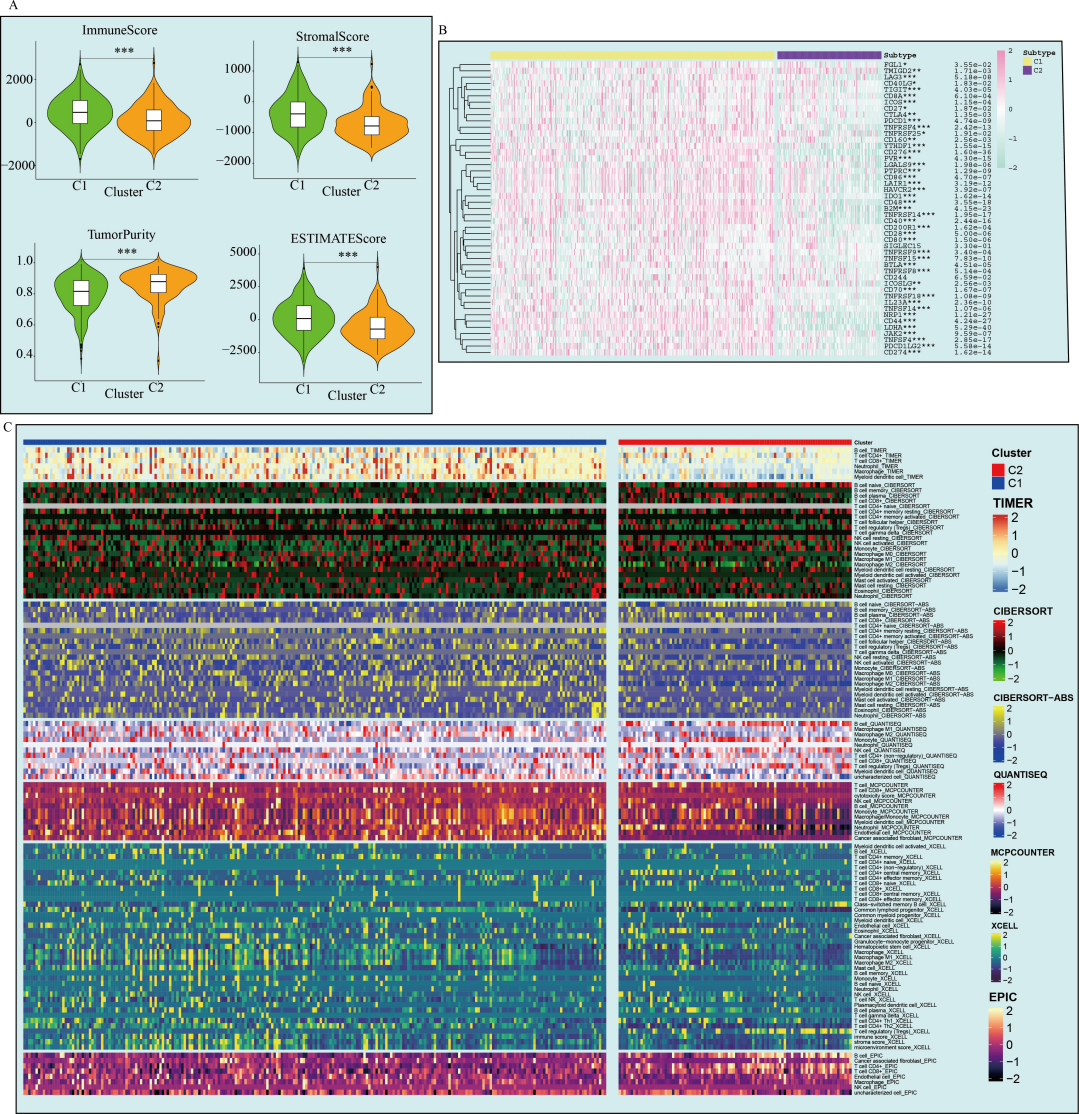
Differences in the immune microenvironment between the two GBM subtypes. **(A)** ESTIMATE analysis of the two subtypes. **(B)** Immune checkpoint expression in the two subtypes. **(C)** Immune cell infiltration analysis of the two subtypes. (*p < 0.05; **p<0.01; ***p<0.001).

This study evaluated the immune microenvironment of the two subtypes using multiple immune infiltration analyses. ESTIMATE analysis showed that C1 had higher matrix scores, immune scores, and ESTIMATE scores compared to C2, while having lower tumor purity, suggesting that C1 has a richer immune microenvironment (**Figure 3A**). However, C1 exhibited significantly higher expression of immune inhibitory molecules (such as PD-L1 and LAG3) compared to C2, indicating that C1 has a more pronounced immune inhibitory immune microenvironment (**Figure 3B**). Based on online analysis using the TIMER database, we applied multiple immune infiltration methods, such as TIMER, CIBERSORT, EPIC, QUANTISEQ, MCPCOUNTER, and XCELL, to analyze the immune cell infiltration in the two subtypes. The results showed that C1 had more immune cell infiltration than C2, especially in multiple T cell subpopulations (**Figure 3C**). Since this study classified tumors based on 82 markers associated with prolif-like T cells, the C1 subtype can be considered a subtype with higher immune cell infiltration. However, this does not necessarily indicate that the C1 subtype has active antitumor immune responses. On the contrary, based on existing results, the C1 subtype exhibits more pronounced immune suppression, which may be due to the increased number of immune cells in the immune microenvironment that promote tumor growth.

### Screening of important key genes for survival prognosis models of GBM

**Figure 4:**
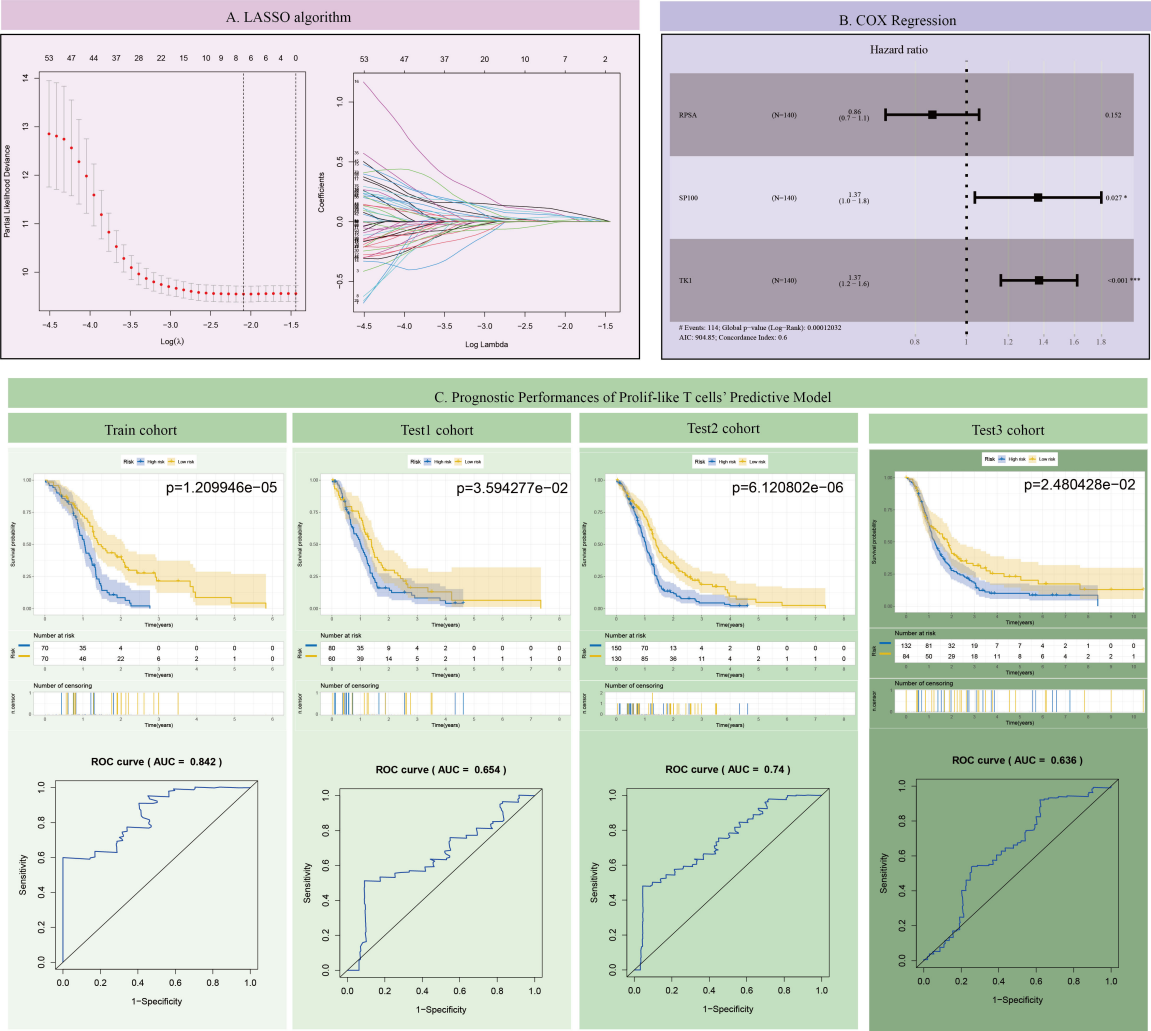
Construction and validation of the GBM survival prognosis model. **(A)** LASSO algorithm. **(B)** COX Regression. **(C)** Prognostic Performances of Prolif-like T cells’ Predictive Model.

This study further screened representative key genes from the 82 markers related to prolif-like T cells and constructed a survival prediction model using the clinical survival data of GBM patients. First, the genes were preliminarily screened through Lasso analysis and training set data (**Figure 4A**). Further, through multi-factor Cox regression analysis, three genes (TK1, SP100, and RPSA) were identified as having survival predictive value, and a survival model and risk scores were constructed. Among these, TK1 exhibited a highly significant high-risk feature (HR = 1.37, p < 0.001) (**Figure 4B**). The survival curves of the training set showed that patients with high-risk scores had poorer outcomes (p = 1.209946e-05). ROC analysis revealed an AUC value of 0.842, confirming the good predictive performance of the survival model (**Figure 4C**). Validation of the survival model using three test sets (test1-3) showed that the survival curve for test1 indicated poorer prognosis in patients with high-risk scores (p=3.594277e-02), and ROC analysis revealed an AUC value of 0.654. The survival curve for test2 showed that patients with high-risk scores had poorer outcomes (p=6.120802e-06), and ROC analysis revealed an AUC value of 0.74. Test 3 survival curves showed that patients with high-risk scores had poorer outcomes (p = 2.480428e-02), and ROC analysis revealed an AUC value of 0.636. In summary, all test sets demonstrated that the survival model has good predictive performance (p < 0.05, AUC > 0.6). This study further analyzed the expression of the three genes in 12 subclusters using scRNA-seq data, and the results showed that only TK1 exhibited significantly elevated expression in prolif-like tumor, prolif-like macrophage, and prolif-like T cell (**Supplementary Figure S1**). Therefore, this study suggests that TK1 is an important proliferative marker in the tumor microenvironment of GBM and has certain research value.

### Functional role of TK1 in the immune microenvironment of GBM

**Figure 5:**
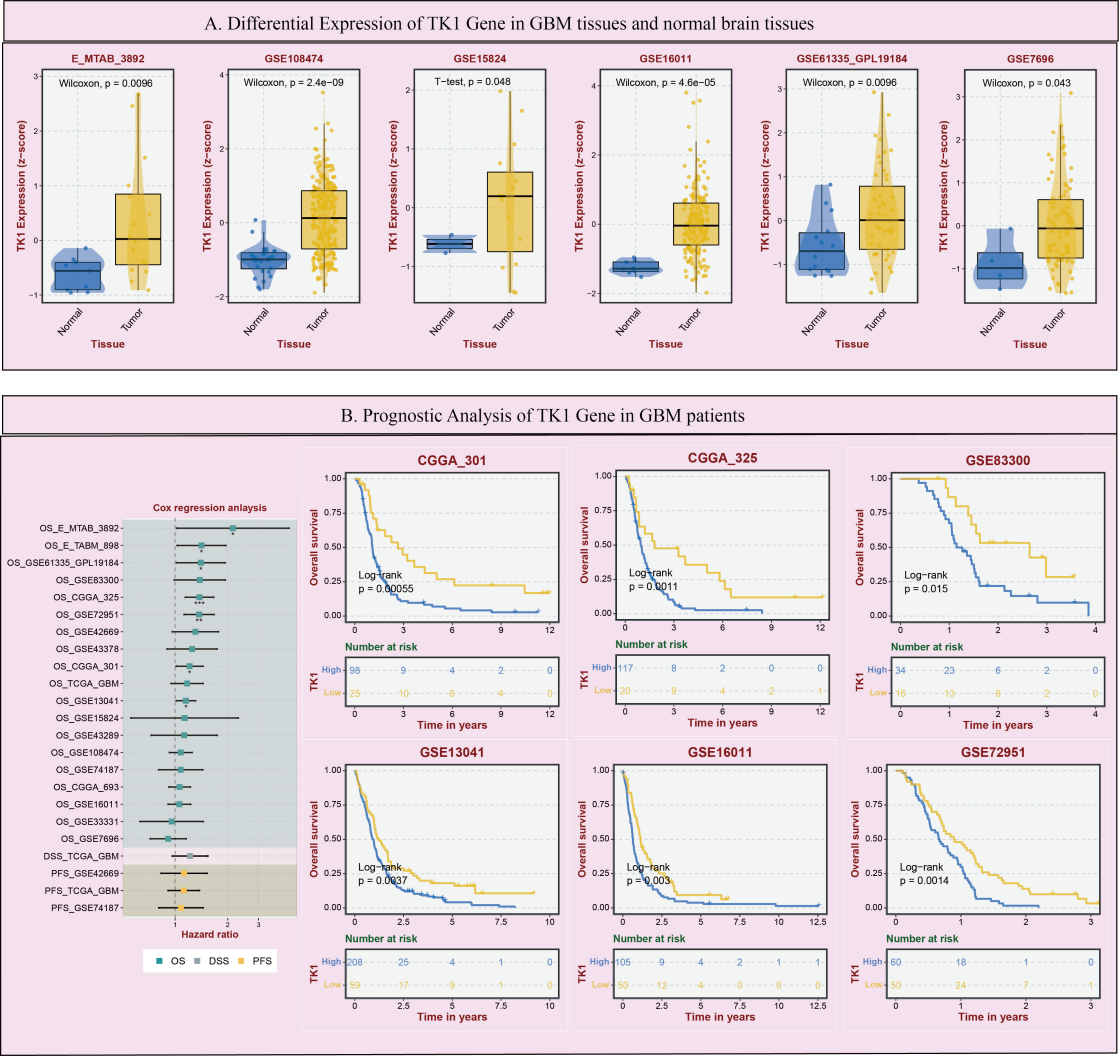
Expression and prognostic impact of TK1 in GBM. **(A)** Differential Expression of TK1 Gene in GBM tissues and normal brain tissues. **(B)** Prognostic Analysis of TK1 Gene in GBM patients. (*p < 0.05; **p<0.01; ***p<0.001).

Using the BEST (Biomarker Exploration of Solid Tumors) online tool, this study further analyzed the expression of TK1 in GBM tumors and its impact on prognosis. In a large number of GBM cohorts, TK1 exhibited higher expression in tumor tissues compared to normal tissues (**Figure 5A**).

Additionally, in multiple GBM survival cohorts, patients with higher TK1 expression tended to have poorer outcomes (**Figure 5B**). Furthermore, based on TCGA pan-cancer cohort analysis, we found that TK1 exhibited increased SNV mutations (**Supplementary Figure S2A**) and mRNA expression (**Supplementary Figure S2B**) in various tumors.

**Figure 6:**
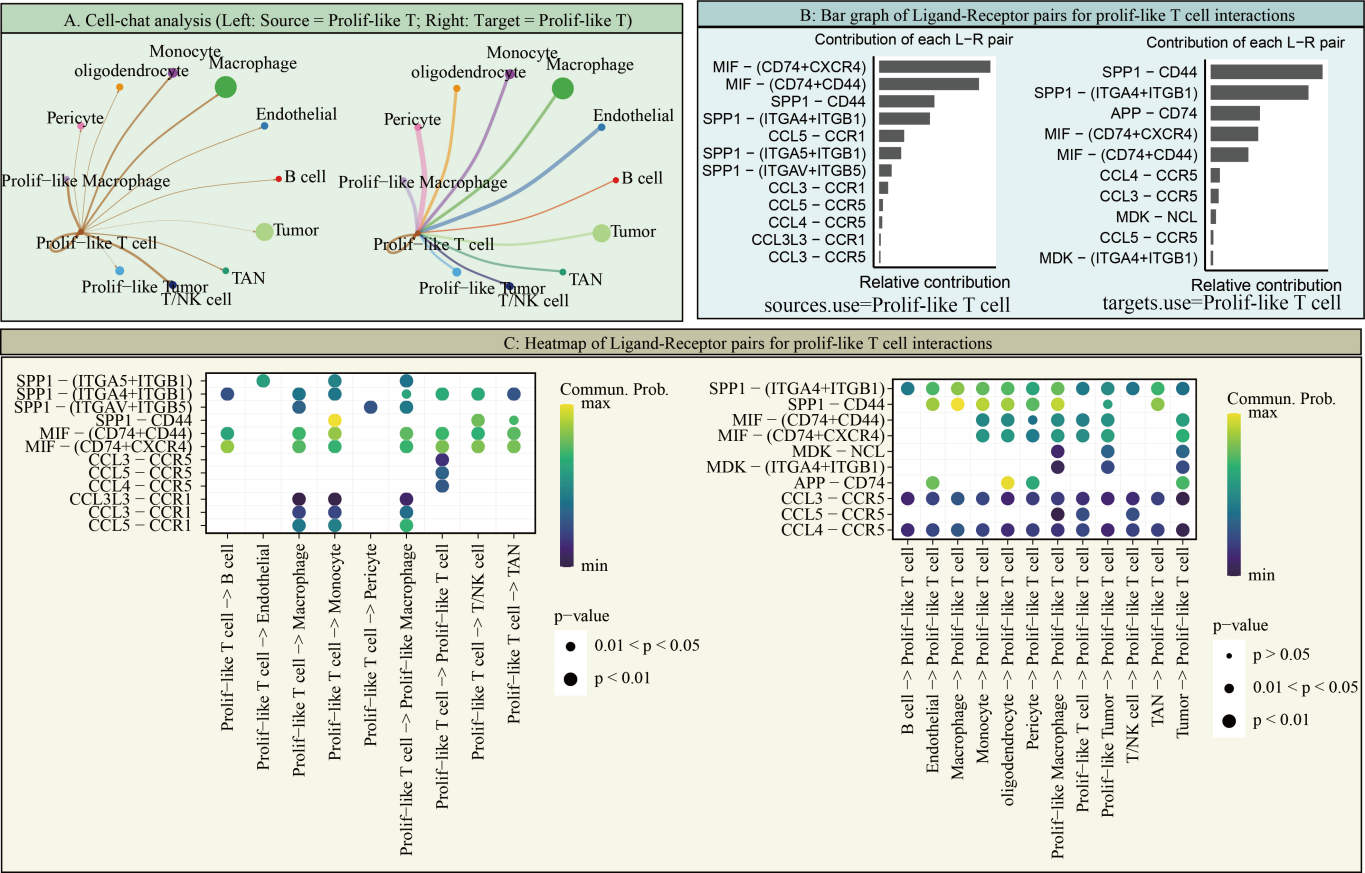
Cell communication of Prolif-like T cells. **(A)** Cell-chat analysis (Left: Source = Prolif-like T; Right: Target = Prolif-like T). **(B)** Bar graph of Ligand-Receptor pairs for Prolif-like T cell interactions. **(C)** Heatmap of Ligand-Receptor pairs for Prolif-like T cell interactions.

**Figure 7:**
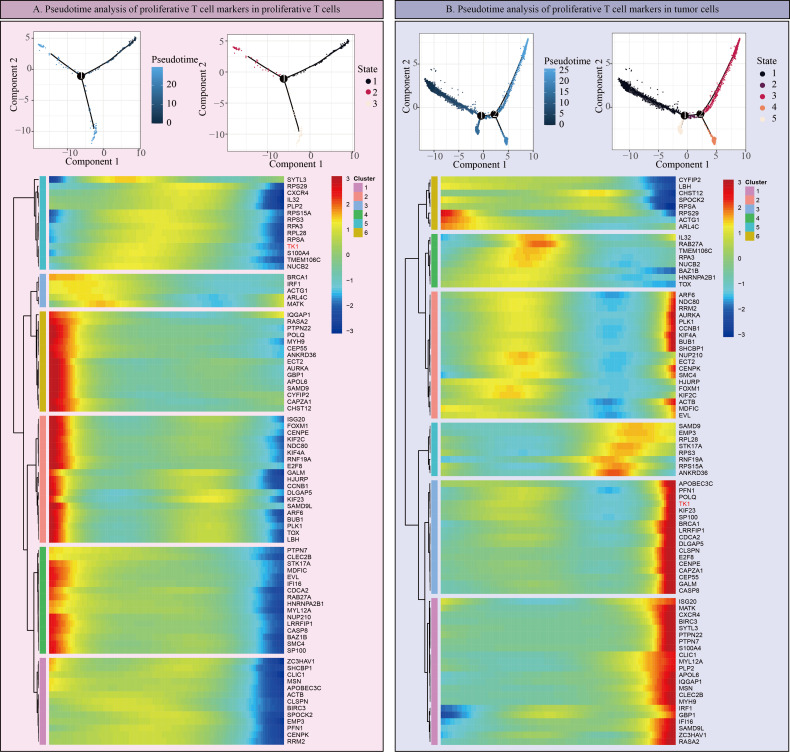
Pseudotime analysis of proliferative T cells and tumors. **(A)** Pseudotime analysis of Prolif-like T cell markers in Prolif-like T cells. **(B)** Pseudotime analysis of Prolif-like T cell markers in tumor cells.

However, it is worth noting that in scRNA-seq data, TK1 expression is not particularly high in prolif-like tumor and tumor, but is predominantly expressed in prolif-like macrophage and prolif-like T cell. We further performed cell communication analysis on scRNA-seq data and found that prolif-like tumor and prolif-like macrophage exhibit increased cell communication with prolif-like T cell (**Figure 6A**). Prolif-like T cells primarily communicate with other cell populations through SPF1, MIF, and chemokine-related signaling pathways (**Figure 6B**). Prolif-like tumor and prolif-like macrophage can communicate with prolif-like T cells through multiple pathways (**Figure 6C**). This suggests that the three subpopulations of prolif-like tumor, prolif-like macrophage, and prolif-like T cells exhibit extensive communication. Furthermore, prolif-like tumor and prolif-like macrophage can suppress T cell activation through cell communication and further promote the pro-inflammatory role of T cells. This study also performed a pseudotime analysis of prolif-like T cells and tumor cells based on scRNA-seq data, analyzing the expression of 82 marker genes during the developmental process of the two cell populations. The results showed that the expression of most genes in prolif-like T cells increased in the early stage and decreased in the late stage (**Figure 7A**). In contrast, the expression of genes in tumor cells decreased in the early stage and increased in the late stage (**Figure 7B**). TK1 also exhibited similar changes in both subpopulations. However, TK1 expression in prolif-like T cells showed a more gradual change, while tumor cells exhibited a sudden increase in TK1 expression in the late stage. Based on these findings, we have reason to believe that tumor cells, and possibly prolif-like tumor cells, may have potential communication via TK1 with prolif-like T cells. More specifically, TK1 in prolif-like T cells enters tumors through some pathway, promoting the transformation of tumors into prolif-like tumors and accelerating tumor growth. Therefore, we believe that inhibiting TK1 expression in tumors and the tumor microenvironment is a potential therapeutic strategy for treating GBM.

### Effects of targeting TK1 on GBM cells

**Figure 8:**
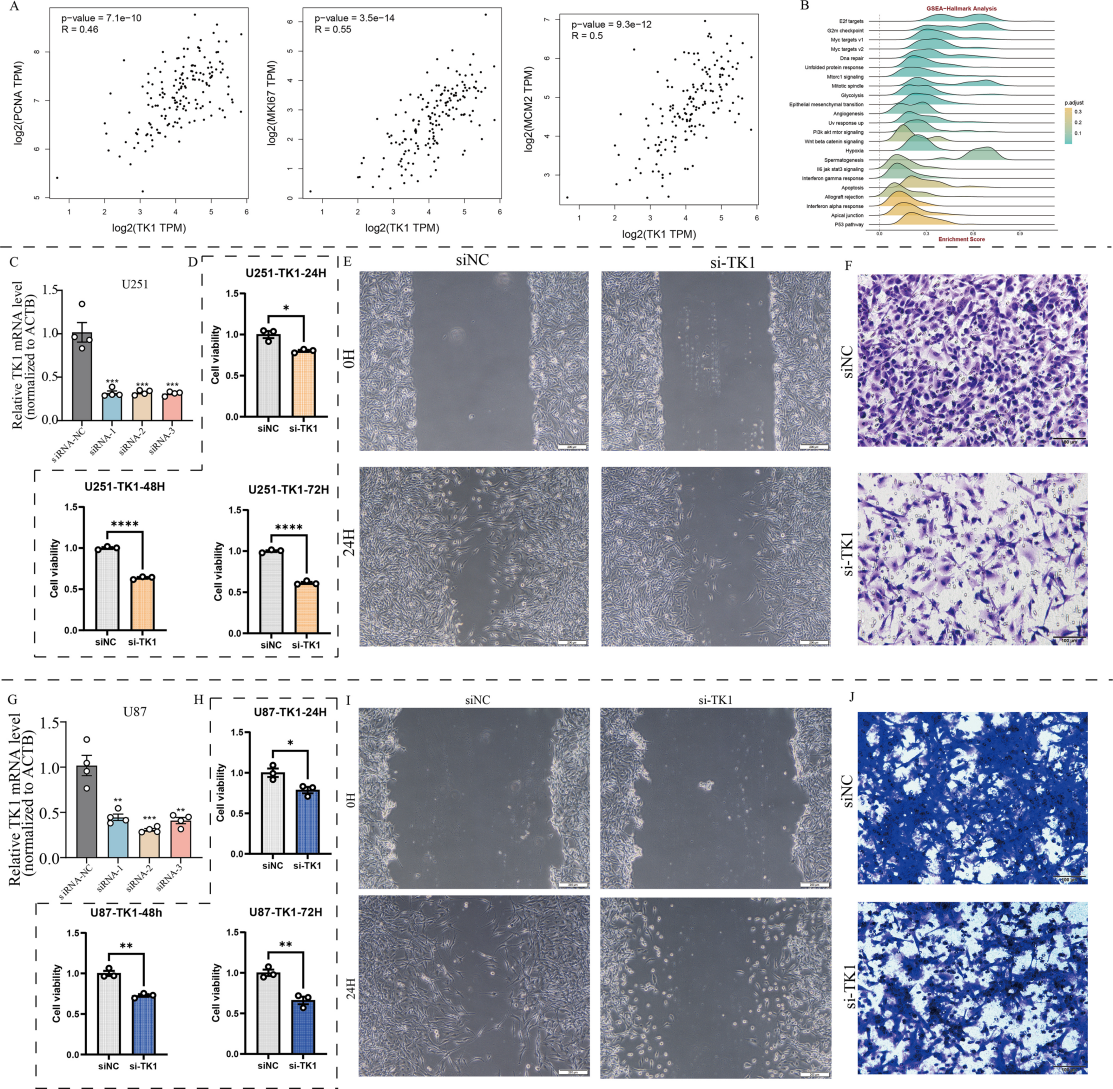
Effects of TK1 on GBM cell function. **(A)** Expression relationship between TK1 and proliferative markers. **(B)** TK1-related pathways based on GSEA-Hallmark analysis. **(C)** Construction of si-TK1 U251 cell lines. **(D)** CCK8 assay of si-TK1 U251 cell lines. **(E)** Cell scratch assay of si-TK1 U251 cell lines. **(F)** Transwell invasion assay of si-TK1 U251 cell lines. **(G)** Establishment of si-TK1 U87 cell lines. **(H)** CCK-8 assay of si-TK1 U87 cell lines. **(I)** Cell scratch assay of si-TK1 U87 cell lines. **(J)** Transwell invasion assay of si-TK1 U87 cell lines. *: p <0.05; **: p < 0.01; ***: p < 0.001; ****: p < 0.0001.

Using the BEST (Biomarker Exploration of Solid Tumors) online tool, we found that TK1 expression in GBM is associated with multiple proliferation marker genes (**Figure 8A**).

**Figure 9:**
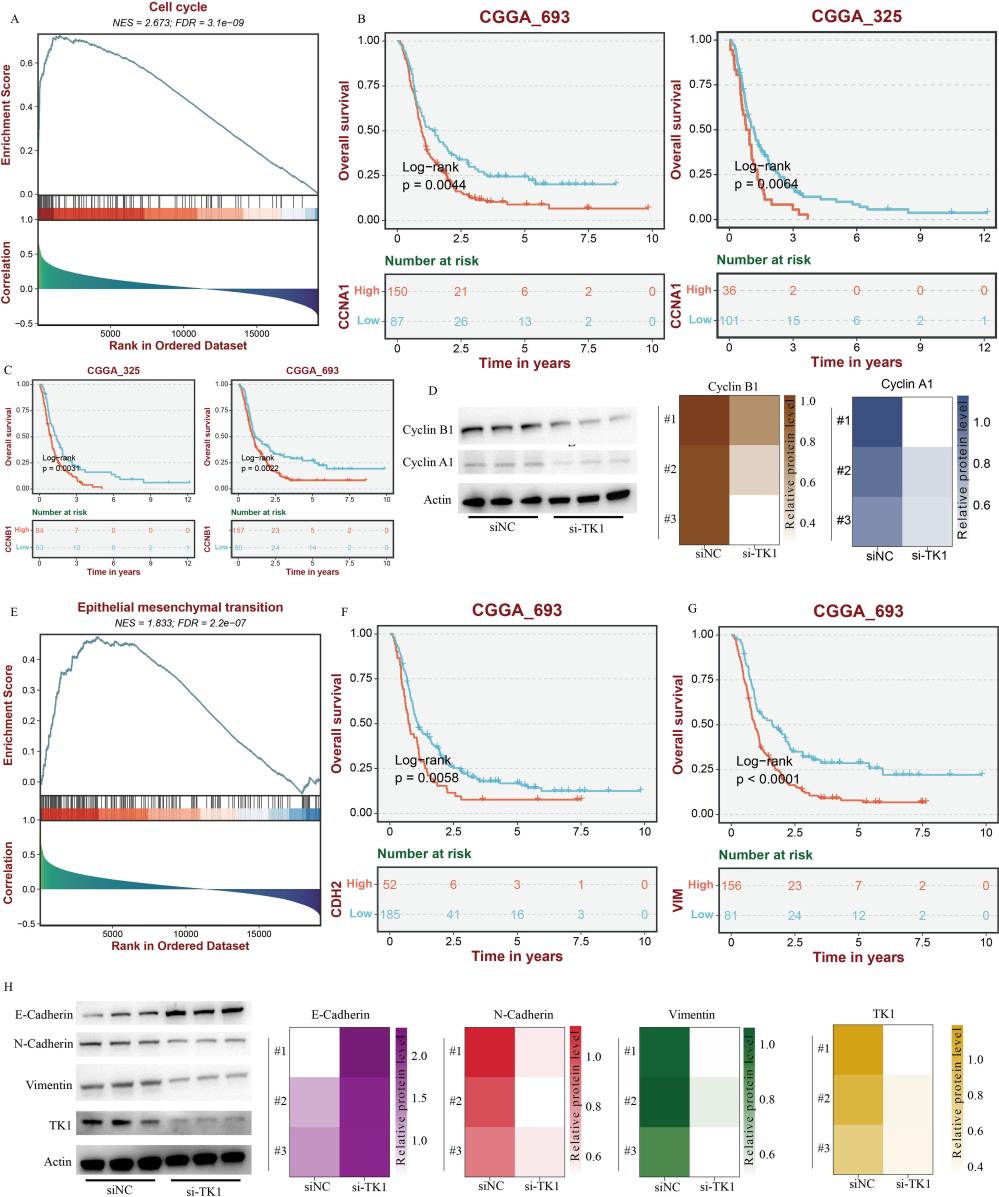
TK1 influences the cell cycle and EMT process in GBM. **(A)** The impact of TK1 on GBM cell cycle based on GSEA analysis. **(B)** Prognostic analysis of CCNA1. **(C)** Prognostic analysis of CCNB1. **(D)** The impact of TK1 on Cyclin A1 and Cyclin B1. **(E)** The impact of TK1 on GBM EMT processing based on GSEA analysis. **(F)** Prognostic analysis of CDH2. **(G)** Prognostic analysis of VIM. **(H)** The impact of TK1 on E-Cadherin, N-Cadherin, and Vimentin.

The results showed that PCNA expression was significantly correlated with TK1 expression (R = 0.46, p = 7.1e-10). MKI67 expression was significantly correlated with TK1 expression (R = 0.55, p = 3.5e-14). MCM2 expression was significantly correlated with TK1 expression (R = 0.5, p = 9.3e-12). Pathway analysis based on Hallmark pathway revealed that TK1 influences the enrichment levels of the cell cycle pathway and the epithelial-mesenchymal transition (EMT) pathway (**Figure 8B**). Further GSEA analysis confirmed that these two pathways are important downstream pathways of TK1 (**Figures 9A, E**).

Meanwhile, there are no effective inhibitors targeting TK1 in clinical practice. Therefore, this study used siRNA to interfere with TK1 expression in GBM cell lines to simulate the therapeutic process. This study primarily utilized two GBM cell lines, U251 and U87, and constructed si-TK1 cell models using the siRNA method (**Figures 8C, G**). CCK8 assay results showed that at 24 h, 48 h, and 72 h, inhibiting TK1 expression reduced the proliferation of both GBM cell lines (**Figures 8D, H**). Cell scratch assay results showed that inhibiting TK1 expression shortened the migration distance of both GBM cell lines (**Figures 8E, I**). Transwell invasion assay results demonstrated that inhibiting TK1 expression suppressed the invasion capacity of both GBM cell lines (**Figures 8F, J**). To further investigate the effects of TK1 inhibition on the cell cycle and EMT, we analyzed the expression of relevant proteins in the si-TK1-treated U251 cell line. The results showed that inhibiting TK1 expression significantly suppressed the expression of CCNA1 (cyclin A1) and CCNB1 (cyclin B1) (**Figure 9D**), and high expression of CCNA1 and CCNB1 in the GBM survival cohort was associated with poorer prognosis (**Figures 9B, C**). Additionally, inhibiting TK1 expression significantly suppressed the expression of CDH2 (N-Cadherin) and VIM (Vimentin) (**Figure 9H**), and high expression of CDH2 and VIM in the GBM survival cohort was associated with poorer prognosis (**Figures 9F, G**). In summary, targeting TK1 inhibition suppresses the cell cycle and EMT process in GBM, suggesting that TK1 is a potential antitumor target in GBM.

### Spatial transcriptomic analysis highlighted the close interconnection among proliferative T cells, tumor cells, EMT signaling, and cell cycle signaling

**Figure 10:**
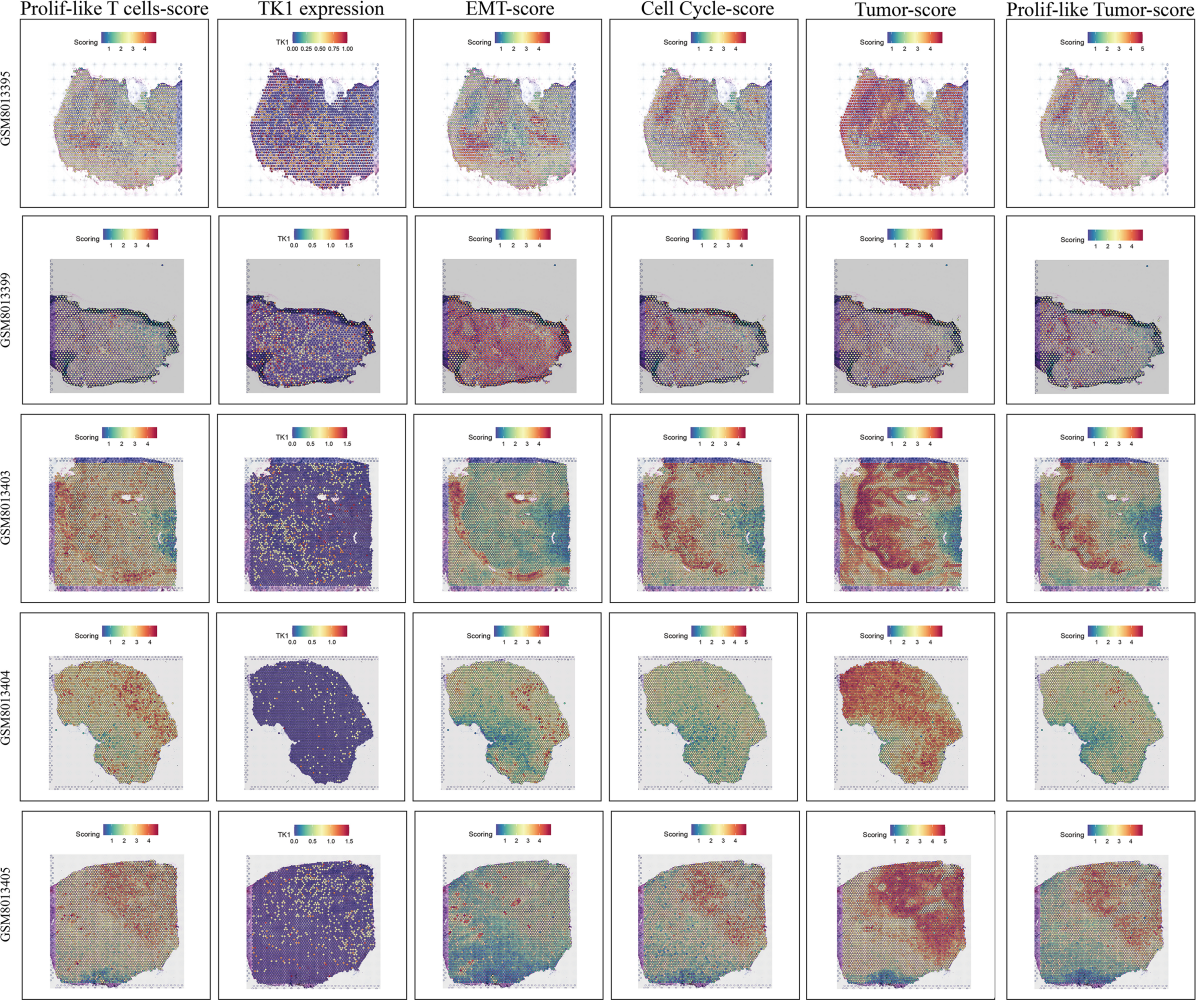
Spatial transcriptomic features revealing the association among proliferative T cells, tumor cells, EMT signaling, and cell cycle signaling.

Spatial transcriptomic analysis (**Figure 10**), from an additional dimension, further emphasized the presence of proliferative T cells, tumor cells, and proliferative tumor cells, as well as the connections among them. Interestingly, we observed that proliferative T cells often appeared in the vicinity of tumor cells, particularly proliferative tumor cells. In these spatial regions, we also detected abnormally active cell cycle and EMT signaling. Notably, the spatial expression pattern of TK1 closely mirrored the distribution of both proliferative T cells and tumor cells. More importantly, the spatial characteristics of TK1 share a similar spatial distribution with EMT signaling and cell cycle signaling. In conclusion, spatial transcriptomics further confirmed the important role of proliferative T cells in GBM, as well as the regulatory capacity of TK1 over cell cycle signaling and EMT signaling.

## Discussion

High-grade gliomas is a collective term for malignant gliomas, referring to gliomas classified as grade III and IV in the World Health Organization (WHO) classification of central nervous system tumors. GBM is the most common and most aggressive subtype of HGG, belonging to grade IV gliomas. GBM is the subgroup with the poorest prognosis among HGG, characterized by high recurrence rates and high drug resistance. Immunotherapy is a potential treatment approach for GBM, and overcoming the inhibitory effects of the GBM tumor microenvironment is a potential therapeutic direction for future GBM treatment. This study found that the immune microenvironment of GBM contained a significant number of Prolif-like T cells, which were highly associated with GBM tumor cells. Some markers associated with Prolif-like T cells are potential factors influencing poor prognosis in GBM. This study identified TK1 as an important marker associated with Prolif-like T cells through multiple bioinformatics methods, which can influence the cell cycle and EMT process of GBM cells and is a potential therapeutic target for GBM.

TK1 is an essential cytoplasmic enzyme involved in DNA synthesis and repair, primarily catalyzing the phosphorylation of thymidine to thymidine monophosphate (dTMP), a key rate-limiting step in the synthesis of DNA precursor molecules. Its activity and expression levels peak during the S phase and G2 phase of the cell cycle, closely correlating with cellular proliferation status. This characteristic makes it an important molecular marker for assessing cellular proliferation activity. Under physiological conditions, TK1 activity is primarily present in tissues with active proliferation, such as bone marrow, lymphoid tissues, intestinal mucosa, and germ cells. However, in pathological conditions, particularly in malignant tumors, due to uncontrolled proliferation of tumor cells, TK1 is synthesized and released into the blood in large quantities, leading to a significant increase in serum TK1 activity or concentration. This phenomenon has been widely confirmed in various solid tumors and hematological malignancies (28–32). Therefore, serum TK1 detection, as a non-invasive liquid biopsy method, demonstrates significant value in the early screening, auxiliary diagnosis, efficacy monitoring, prognosis assessment, and recurrence risk prediction of tumors. This study found that high expression of TK1 in GBM tumor tissues was also an important indicator of poor tumor prognosis.

TK1 is a key enzyme in the DNA nucleotide salvage synthesis pathway and is closely associated with the cell cycle. In normal cells, TK1 activity exhibits strict cell cycle dependence, primarily expressed during the S phase and rapidly degraded after cell division, with extremely low levels in serum. However, in malignant tumors, this precise regulation is significantly disrupted. The abnormal proliferative characteristics of tumor cells lead to a large number of cells entering the S phase and maintaining high expression of TK1; simultaneously, increased apoptosis, necrosis, and tumor vascular permeability in tumor tissues collectively promote the massive release of TK1 protein or its fragments into the bloodstream, making it an important serum marker reflecting abnormal proliferative activity of tumor cells (33). Its overexpression is not only associated with dysregulation of cell cycle regulatory factors but also involves abnormal activation of multiple tumor-related signaling pathways and changes in epigenetic modifications (34).

Recent studies have increasingly revealed that TK1 is not only a marker of tumor cell proliferation but also a key factor actively involved in shaping the immuno-suppressive tumor microenvironment (TME), influencing the composition and functional state of the TME through multiple mechanisms. High expression of TK1 in tumor cells accelerates dTTP synthesis, directly meeting the DNA synthesis requirements for rapid proliferation, thereby promoting tumor growth and invasion. Notably, tumor cells can release TK1 protein or its active forms into the TME via the exosome pathway. These exosomal TK1 (exTK1) molecules are taken up by immune cells such as tumor-associated macrophages (TAMs), MDSCs, and dendritic cells (DCs), significantly disrupting their normal immune functions. For example, exTK1 can suppress T cell activation and proliferation, induce T cell apoptosis or exhaustion, promote TAMs to polarize toward the immuno-suppressive M2 phenotype, and enhance the immuno-suppressive activity of MDSCs, collectively leading to impaired effector T cell function and creating an immuno-suppressive environment conducive to tumor escape (35). TK1 may activate pro-inflammatory and pro-survival pathways such as NF-κB and STAT3 by influencing key metabolite levels or through direct signal transduction. These pathways not only further promote tumor cell proliferation, survival, and invasion but also continuously stimulate the production of immuno-suppressive cytokines and the recruitment and activation of inhibitory immune cells in the TME, forming a positive feedback loop that maintains immunosuppression and pro-tumor inflammation (36). Furthermore, TK1 expression is associated with tumor-infiltrating lymphocytes, immune subtypes, and immune regulatory factors in most cancers (37, 38). In summary, the role of TK1 in tumors extends beyond that of a simple cell proliferation marker. It profoundly and extensively participates in shaping the immune-suppressive and tumor-promoting TME by promoting tumor cell self-proliferation, mediating exosome-dependent immune suppression, and driving pro-tumor inflammatory signaling pathways. Therefore, targeting TK1 or its mediated signaling pathways (such as developing TK1 inhibitors or targeting exosomes carrying TK1) not only aims to inhibit tumor cell proliferation itself but also holds potential for reshaping the TME, reversing immune suppression, and enhancing antitumor immune responses. This study found that TK1 also plays an important role in the tumor microenvironment of GBM, serving as an important marker associated with prolif-like T cells, with elevated expression not only in prolif-like T cells but also in prolif-like Tumors. Furthermore, the high expression of TK1 in GBM tumor tissues is an indicator of poor tumor prognosis and a potential feature of tumor progression. Our study also found that tumor cell clusters in single-cell data did not exhibit high expression of TK1. Therefore, based on multiple analysis results and previous studies, we speculate that there is TK1 communication between tumor cells and Prolif-like T cells, which facilitates further proliferation and progression of tumor cells. Therefore, this study identified targeting TK1 (both intracellular and extracellular are potential targets) as a potential therapeutic strategy for treating GBM. However, there are currently no available TK1 inhibitors. Developing TK1 inhibitors and specific treatment pathways remains an important research direction. While our study provides a foundation for TK1 research in GBM, further mechanistic studies are needed to elucidate the TK1 communication between Tumor and Prolif-like T cells in GBM.

## Conclusions

In summary, the high infiltration of prolif-like T cells is closely associated with poor prognosis in GBM. TK1, as one of their major markers, can promote GBM proliferation, migration, and invasion. Targeting TK1 may represent a new avenue of hope for patients with GBM.

## Data Availability

The original contributions presented in the study are included in the article/**Supplementary Material**. Further inquiries can be directed to the corresponding author.

## Supplementary material

Supplementary Figure 1 Model gene expression in various cell types in scRNA-seq data.

Supplementary Figure 2 Pan-cancer features of Prolif-like T markers in the TCGA pan-cancer cohort. **(A)** SNV analysis. **(B)** mRNA expression analysis.
