# Supplementary figures and images for: Thymidine kinase 1 related to Prolif-like T cells promoted GBM through regulation of cell cycle and EMT signals: a comprehensive research based on multi-omics analysis and experimental validation
Source: Front Immunol. 2025 Sep 25;16:1655980. doi: 10.3389/fimmu.2025.1655980 (PMC12507609; doi:10.3389/fimmu.2025.1655980)

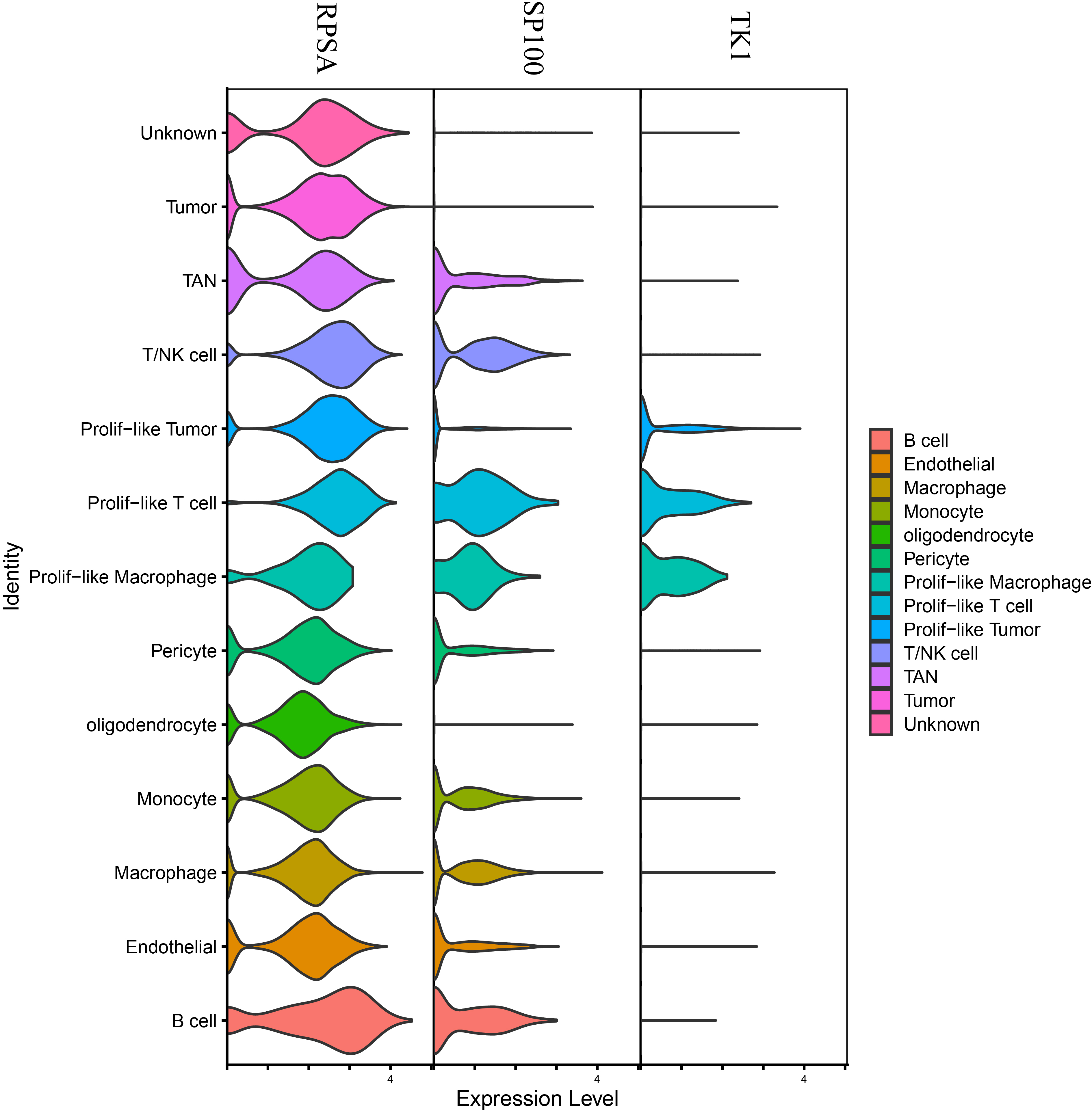

Supplement: Supplementary Figure 1 — Model gene expression in various cell types in scRNA-seq data. [file Image1.jpeg]

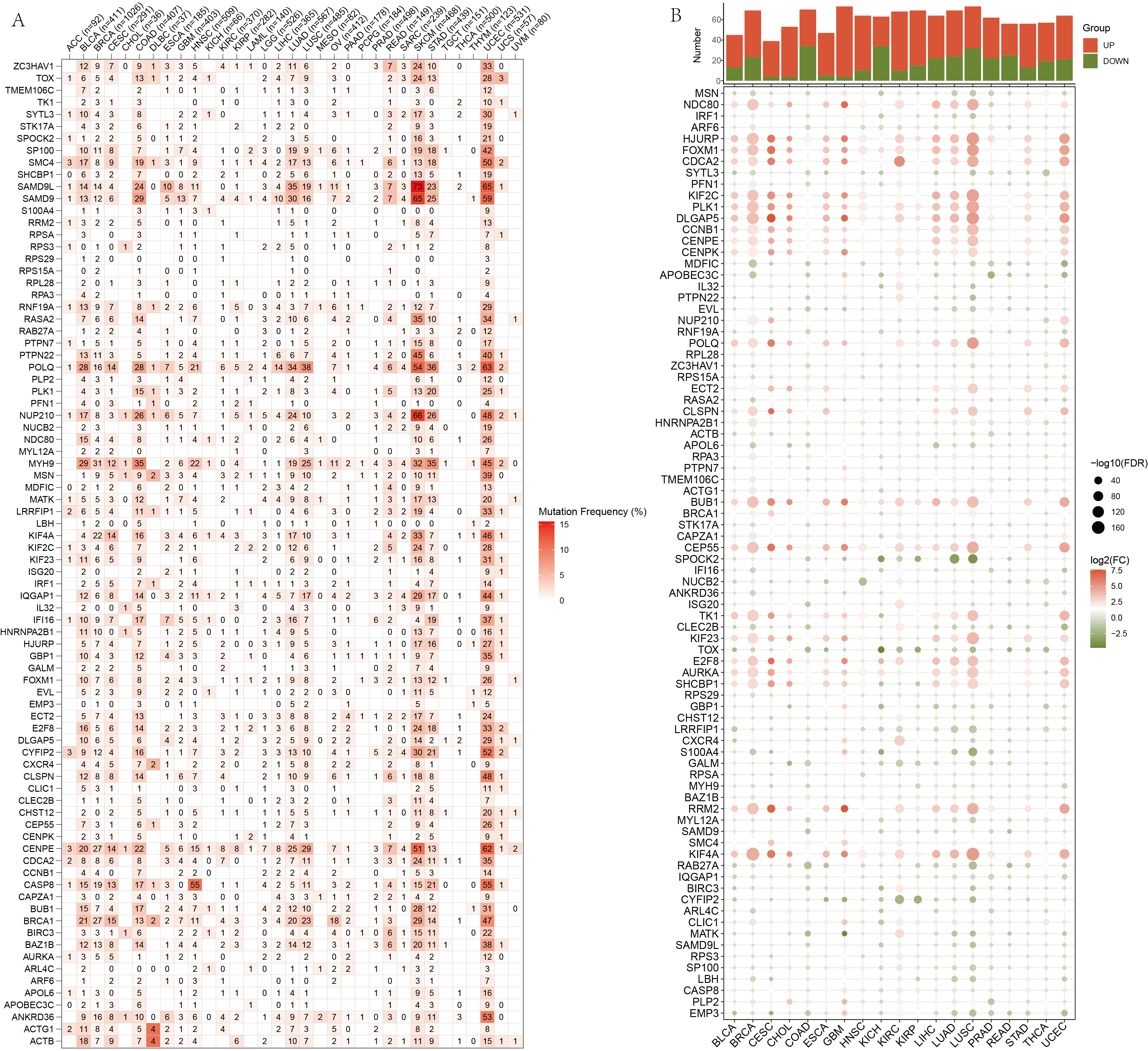

Supplement: Supplementary Figure 2 — Pan-cancer features of Prolif-like T markers in the TCGA pan-cancer cohort. (A) SNV analysis. (B) mRNA expression analysis. [file Image2.jpeg]
